# Malignant Pleural Mesothelioma Interactome with 364 Novel Protein-Protein Interactions

**DOI:** 10.3390/cancers13071660

**Published:** 2021-04-01

**Authors:** Kalyani B. Karunakaran, Naveena Yanamala, Gregory Boyce, Michael J. Becich, Madhavi K. Ganapathiraju

**Affiliations:** 1 Supercomputer Education and Research Centre, Indian Institute of Science, Bangalore 560012, India; kalyanik@iisc.ac.in; 2 Exposure Assessment Branch, National Institute of Occupational Safety and Health, Center for Disease Control, Morgantown, WV 26506, USA; yanamala.naveena@gmail.com (N.Y.); omu0@cdc.gov (G.B.); 3 Department of Biomedical Informatics, School of Medicine, University of Pittsburgh, Pittsburgh, PA 15206, USA; becich@pitt.edu; 4 Intelligent Systems Program, School of Computing and Information, University of Pittsburgh, Pittsburgh, PA 15213, USA

**Keywords:** malignant mesothelioma, protein-protein interactions, systems biology, network analysis, drug repurposing

## Abstract

**Simple Summary:**

Internal organs like the heart and lungs, and body cavities like the thoracic and abdominal cavities, are covered by a thin, slippery layer called the mesothelium. Malignant pleural mesothelioma (MPM) is an aggressive cancer of the lining of the lung, where genetics and asbestos exposure play a role. It is not diagnosable until it becomes invasive, offering only a short survival time to the patient. To help understand the role of the genes that relate to this disease most of which are poorly understood, we constructed the ‘MPM interactome’, including in it the protein-protein interactions that we predicted computationally and those that are previously known in the literature. Five novel protein-protein interactions (PPIs) were tested and validated experimentally. 85.65% of the interactome is supported by genetic variant, transcriptomic, and proteomic evidence. Comparative transcriptome analysis revealed 5 repurposable drugs targeting the interactome proteins. We make the interactome available on a freely accessible web application, Wiki-MPM.

**Abstract:**

Malignant pleural mesothelioma (MPM) is an aggressive cancer affecting the outer lining of the lung, with a median survival of less than one year. We constructed an ‘MPM interactome’ with over 300 computationally predicted protein-protein interactions (PPIs) and over 2400 known PPIs of 62 literature-curated genes whose activity affects MPM. Known PPIs of the 62 MPM associated genes were derived from Biological General Repository for Interaction Datasets (BioGRID) and Human Protein Reference Database (HPRD). Novel PPIs were predicted by applying the HiPPIP algorithm, which computes features of protein pairs such as cellular localization, molecular function, biological process membership, genomic location of the gene, and gene expression in microarray experiments, and classifies the pairwise features as interacting or non-interacting based on a random forest model. We validated five novel predicted PPIs experimentally. The interactome is significantly enriched with genes differentially ex-pressed in MPM tumors compared with normal pleura and with other thoracic tumors, genes whose high expression has been correlated with unfavorable prognosis in lung cancer, genes differentially expressed on crocidolite exposure, and exosome-derived proteins identified from malignant mesothelioma cell lines. 28 of the interactors of MPM proteins are targets of 147 U.S. Food and Drug Administration (FDA)-approved drugs. By comparing disease-associated versus drug-induced differential expression profiles, we identified five potentially repurposable drugs, namely cabazitaxel, primaquine, pyrimethamine, trimethoprim and gliclazide. Preclinical studies may be con-ducted in vitro to validate these computational results. Interactome analysis of disease-associated genes is a powerful approach with high translational impact. It shows how MPM-associated genes identified by various high throughput studies are functionally linked, leading to clinically translatable results such as repurposed drugs. The PPIs are made available on a webserver with interactive user interface, visualization and advanced search capabilities.

## 1. Introduction

Internal organs such as heart and lung, and body cavities such as thoracic and abdominal cavities, are covered by a thin slippery layer of cells called the “mesothelium”. This protective layer prevents organ adhesion and plays a number of important roles in inflammation and tissue repair [1]. The mesothelia that line the heart, lung and abdominal cavity are called pericardium, pleura and peritoneum, respectively. Mesothelioma is the cancer that originates from this lining (described in detail in a recent review article [2]). Most types of mesothelioma metastasize to different locations in the body [3]. Pleural mesotheliomas account for ~90% of malignant mesotheliomas and have a short median survival, of less than 1 year [4].

Malignant pleural mesothelioma (MPM) is associated with exposure to asbestos; it has a long latency period after exposure and is conclusively diagnosable only after reaching the invasive phase [3]. It tends to cluster in families and occurs only in a small fraction of the population exposed to asbestos, suggesting the involvement of a genetic component [5]. These factors necessitate expeditious discovery of genetic predispositions, molecular mechanisms and therapeutics for the disease.

The molecular mechanisms of disease are often revealed by the protein-protein interactions (PPIs) of disease-associated genes. For example, the involvement of transcriptional deregulation in MPM pathogenesis was identified through mutations detected in *BAP1* and its interactions with proteins such as *HCF1*, *ASXL1*, *ASXL2*, *ANKRD1*, *FOXK1* and *FOXK2* [6]. PPI of *BAP1* with *BRCA1* was central to understanding the role of *BAP1* in growth-control pathways and cancer; *BAP1* was suggested to play a role in *BRCA1* stabilization [7,8]. Studies on *BAP1* and *BRCA1* later led to clinical trials of the drug vinorelbine as a second line therapy for MPM patients, and the drug was shown to have rare or moderate effects in MPM patients [9,10]. *BAP1* expression was shown to be necessary for vinorelbine activity; 40% of MPM patients in a study showed low *BRCA1* expression and vinorelbine resistance [11,12,13]. Further, 60% of the disease-associated missense mutations perturb PPIs in human genetic disorders [14].

Despite their importance, only about 10–15% of expected PPIs in the human protein interactome are currently known; for nearly half of the human proteins, not even a single PPI is currently known [15]. Due to the sheer number of PPIs remaining to be discovered in the human interactome, it becomes imperative that biological discovery be accelerated by computational and high-throughput biotechnological methods. We developed a computational model, called HiPPIP (high-precision protein-protein interaction prediction) that is deemed accurate by computational evaluations and experimental validations of 18 predicted PPIs, where all the tested pairs were shown to be true PPIs ([16,17] and current work, and other unpublished works). HiPPIP computes features of protein pairs such as cellular localization, molecular function, biological process membership, genomic location of the gene, and gene expression in microarray experiments, and classifies the pairwise features as interacting or non-interacting based on a random forest model [16]. Though each of the features by itself is not an indicator of an interaction, a machine learning model was able to use the combined features to make predictions with high precision. The threshold of HiPPIP to classify a protein-pair as “a PPI” was set high in such a way that it yields very high-precision predictions, even if low recall. Novel PPIs predicted using this model are making translational impact. For example, they highlighted the role of cilia and mitochondria in congenital heart disease [18,19], that oligoadenylate synthetase-like protein (*OASL*) activates host response during viral infections through RIG-I signaling via its PPI with retinoic acid-inducible gene I (*RIG-I*) [17], and led to the identification of drugs potentially repurposable for schizophrenia [20], one of which is currently under clinical trials.

In this work, we studied MPM-associated genes and their PPIs assembled with HiPPIP and analyzed the MPM interactome to draw translatable results. We demonstrate the various ways in which systems-level analysis of this interactome could lead to biologically insightful and clinically translatable results. We made the interactome available to the cancer biology research community on a webserver with comprehensive annotations, so as to accelerate biomedical research on MPM.

## 2. Results

**Figure 1:**
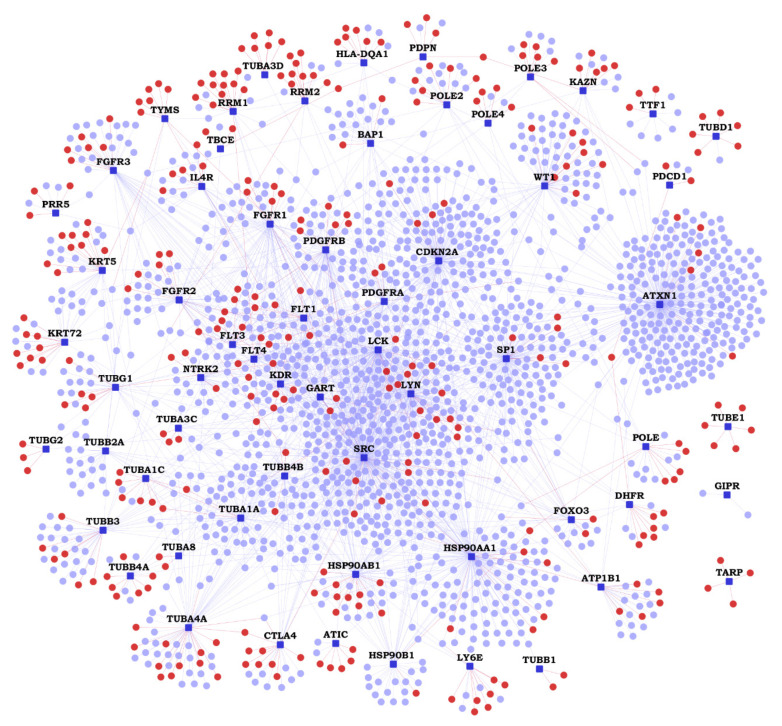
Malignant pleural mesothelioma (MPM) Protein-Protein Interactome: Network view of the MPM interactome is shown as a graph, where genes are shown as nodes and protein-protein interactions (PPIs) as edges connecting the nodes. MPM-associated genes are shown as dark blue square-shaped nodes, novel interactors and known interactors as red and light blue colored circular nodes respectively. Red edges are the novel interactions, whereas blue edges are known interactions.

**Figure 2:**
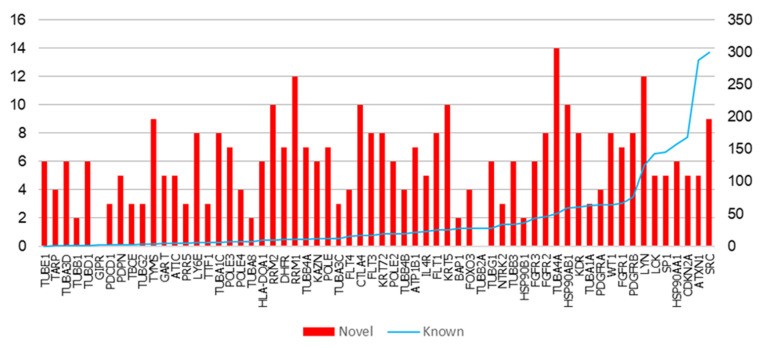
Number of protein-protein interactions (PPIs) in the malignant pleural mesothelioma (MPM) Interactome: The 62 MPM genes are shown along the X-axis, arranged in ascending order of their number of known PPIs. Blue line, right-side axis: Number of known PPIs is shown. Red bars, left-side axis: Number of novel PPIs.

We collected 62 MPM-associated genes from the Ingenuity Pathway Analysis (IPA) suite, which will be referred to as ‘MPM genes’ here; these genes have been reported to affect MPM through gene expression changes or genetic variants, or by being targeted by drugs clinically active against MPM (see details in Data File S1) [21]. Previously known PPIs of the 62 MPM genes were collected from Human Protein Reference Database (HPRD), version 9 [22] and Biological General Repository for Interaction Datasets (BioGRID) version 4.3.194 [23]. Novel (hitherto unknown) PPIs were predicted with HiPPIP, a computational model. We discovered 364 novel PPIs of MPM genes (Table 1), which are deemed highly accurate according to prior evaluation of the HiPPIP model including experimental validations [16]. The MPM interactome thus assembled has 2459 known PPIs and 364 novel PPIs among the 62 MPM-associated genes and 1911 interactors (Figure 1 and Data File S2). Nearly half of the MPM genes had 10 or less known PPIs each, and about 130 novel PPIs have been predicted for these (Figure 2). HiPPIP predicted 920 PPIs of which 556 PPIs were previously known, leaving 364 PPIs to be considered as novel PPIs of the MPM genes. There were an additional 1903 PPIs that are known and not predicted by HiPPIP. This is as expected because the HiPPIP prediction threshold has been fixed to achieve *high precision* by compromising *recall*, which is required for adoption into biology; in other words, it is set to predict only a few PPIs out of the hundreds of thousands of unknown PPIs, but those that are predicted will be highly accurate. It has to be noted that neither PPI prediction nor high throughput PPI screening can be performed with high-precision *and* high-recall. Co-immunoprecipitation (Co-IP) based methods show high-precision and extremely-low recall (detecting only one PPI at a time), whereas multi-screen high-quality yeast 2-hybrid methods show high-precision with low recall (detecting a few tens of thousands of PPIs). Thus, HiPPIP is on par with other methods in terms of precision and the number of new PPIs detected. 18 novel PPIs predicted by HiPPIP were validated to be true (validations have been reported in [16,17], the current work and other unpublished works); the experiments were carried out by diverse research labs.

### 2.1. Experimental Validation of Selected Protein-Protein Interactions (PPIs)

**Figure 3:**
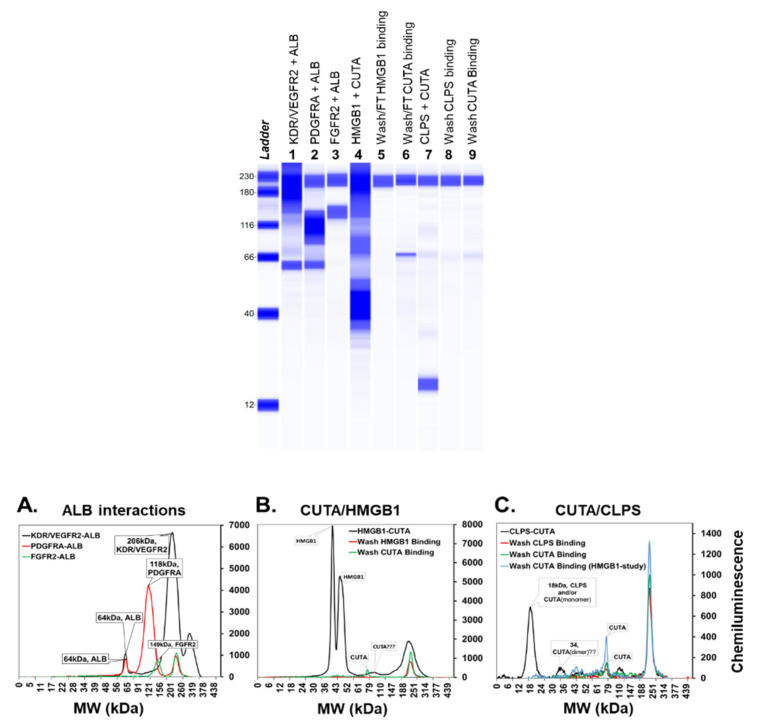
Validation of predicted *ALB* interactions and *CUTA* interactions using Wes™ Simple Western total protein detection assay: Pseudo-gel or virtual-blot like image of the validated interactions of *ALB* (lanes 1–2) and *CUTA* (lanes 4, 7) along with negative control (lane 3). In addition to the final pull-down samples, wash and/or flow through after binding ‘bait’ and ‘prey’ proteins for the *CUTA* interactions are also shown (lanes 5, 6, 8 and 9). The electro-pherogram image of Simple Western results using Total protein size-based assay. (**A**) *ALB* interactions with true positives *KDR*/*VEGFR2*, *PDGFRA* and false positive *FGFR2*. (**B**) *CUTA* interactions with *HMGB1*. (**C**) *CUTA* interactions with *CLPS*. An overlay of the electro-pherogram of the wash from *HMGB1* after *CUTA* binding is also shown in (**C**) for comparison.

We carried out experimental validations of five predicted PPIs chosen for their biological relevance and proximity to MPM genes, namely, *BAP1*-*PARP3*, *KDR*-*ALB*, *PDGFRA*-*ALB*, *CUTA*-*HMGB1* and *CUTA*-*CLPS*. They were validated using protein pull-down followed by protein identification using mass spectrometry (Table S1) or size-based protein detection assay (Figure 3). Each bait protein was also paired with a random prey protein serving as control (specifically, *BAP1*-phospholambin, *ALB*-*FGFR2* and *CUTA*-*FGFR2*). All predicted PPIs were validated to be true, while control pairs tested negative. In addition to these five, another PPI from the MPM interactome, namely *HMGB1*-*FLT1* was validated in our prior work through co-immunoprecipitation [16]. Three novel PPIs, namely *HLA-DQA1*—*HLA-DQB1*, *FGFR2*—*FGF2* and *CDKN2A*—*CDKN2B*, that we reported in the preprint of this work [24], have since been reported as known PPIs in a recent version of BioGRID (downloaded February 2021); these three are treated as known PPIs in the remaining description.

### 2.2. Functional Interactions of Malignant Pleural Mesothelioma (MPM) Genes with Predicted Novel Interactors

We used ReactomeFIViz [25], a Cytoscape plugin, to extract known functional interactions between MPM-associated genes and their novel interactors. Seven novel PPIs had such functional interactions, namely (MPM genes are shown in bold), ***PDGFRB***-*RAPGEF1* (‘*part of the same complex*’, ‘*bound by the same set of ligands*’), ***SP1***→*HNRNPA1* (‘*expression regulation*’), ***HLA-DQA1***→*HLA-DPB1*, *HLA-DQA2*→***HLA-DQA1*** (‘*part of the same complex*’, ‘*catalysis*’), ***CTLA4***-*CD28*, ***PDGFRB***-*PLAUR* (‘*bound by the same set of ligands*’) and ***FGFR2***-*MDM2* (‘*ubiquitination*’).

### 2.3. Web Server

We made the MPM interactome available on a webserver called *Wiki-MPM* (http://severus.dbmi.pitt.edu/wiki-MPM). It has advanced-search capabilities, and presents comprehensive annotations, namely Gene Ontology, diseases, drugs and pathways, of the two proteins of each PPI side-by-side. Here, a user can query for results such as “PPIs where one protein is involved in mesothelioma and the other is involved in immunity”, and then see the results with the functional details of the two proteins side-by-side. The PPIs and their annotations also get indexed in major search engines like Google and Bing; thus a user searching for ‘*KDR* and response to starvation’ would find the PPIs *KDR*-*CAV1* and *KDR*-*ALB*, where the interactors are each involved in ‘response to starvation’. Querying by biomedical associations is a unique feature which we developed in Wiki-Pi that presents known interactions of all human proteins [26]. Wiki-MPM is a specialized version for disseminating the MPM interactome with its novel PPIs, visualizations and browse features. The novel PPIs have a potential to accelerate biomedical discovery in mesothelioma and making them available on this web server brings them to the biologists in an easily-discoverable and usable manner. Wiki-MPM will be integrated into the National Mesothelioma Virtual Bank [27,28], and will be available to the mesothelioma research community as part of our translational support of cancer research.

### 2.4. Pathway Analysis

**Figure 4:**
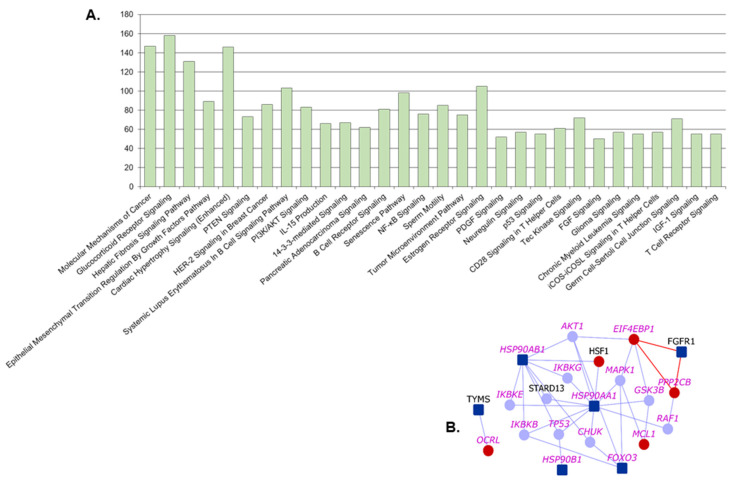
Pathways associated with malignant pleural mesothelioma (MPM) interactome: (**A**) Number of genes from MPM interactome associated with various pathways are shown. Top 30 pathways based on significance of association with the interactome are shown. (**B**) PI3K/AKT Signaling Pathway: Dark blue nodes are MPM genes, light blue nodes are known interactors and red nodes are novel interactors. Nodes with purple labels are genes involved in the PI3K/AKT signaling pathway.

We compiled the list of pathways that any of the proteins of MPM interactome are associated with, using Ingenuity Pathway Analysis suite [29]. Top 30 pathways by statistical significance of association are shown in Figure 4A. A number of pathways such as *NF-κB signaling*, *PI3/AKT signaling*, *VEGF signaling* and *natural killer cell signaling,* are highly relevant to mesothelioma etiology. They are found to be connected to MPM genes through novel PPIs that were previously unknown. For example, the PI3K/AKT signaling pathway regulating the cell cycle is aberrantly active in MPM, and the mesothelioma gene *FGFR1* is connected to this pathway via its novel predicted PPIs with *EIF4EBP1* and *PRP2CB* (Figure 4B) [30]. Statistical significance of association to the interactome, and various MPM genes and novel interactors belonging to these pathways are shown in Table 2 and Data File S3. A cancer biologist may utilize the Supplementary Data (Data Files S2 and S3) to study novel PPIs that connect MPM genes to a pathway that they are interested in studying.

### 2.5. Potentially Repurposable Drugs

**Figure 5:**
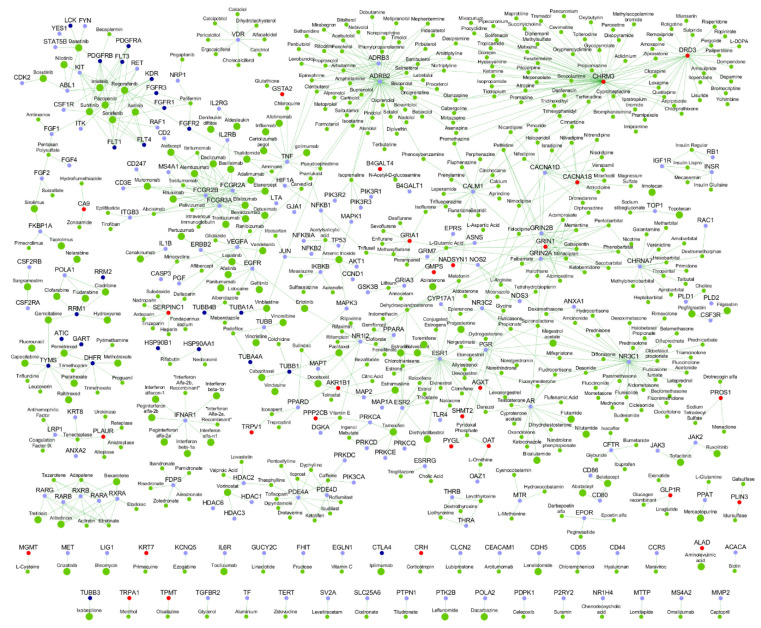
Malignant pleural mesothelioma (MPM) Drug-Protein Interactome: The network shows the drugs (green color nodes) that target the proteins in the MPM interactome. Larger green nodes correspond to drugs that target the anatomic category ‘antineoplastic and immunomodulating agents’. The color legend for genes (proteins) is as shown in Figure 1, with MPM genes in dark blue, their known interactors in light blue and novel interactors in red.

We previously identified drugs potentially repurposable for schizophrenia through interactome analysis, and one of them is currently in clinical trials (ClinicalTrials.gov Identifier: NCT03794076) and another clinical trial has been funded and is yet to start [20]. Following this methodology, we constructed the MPM drug-protein interactome that shows the drugs that target any protein in the MPM interactome. This analysis has been carried out on an earlier version of BioGRID (3.4.159), which had fewer known PPIs, as reported in the preprint version of the paper [24], and has not been recomputed with the latest version of BioGRID unlike the other analyses presented here. There are 513 unique drugs that target 206 of these proteins (of which 28 are novel interactors that are targeted by 147 drugs) (Figure 5 and Data File S4). We adopted the established approach of comparing drug-induced versus disease-associated differential expression using the BaseSpace correlation software (previously called NextBio) [31,32], to identify five drugs that could be potentially repurposable for MPM (Table 3; the table also shows corresponding information for two known MPM drugs). These are: *cabazitaxel*, used in the treatment of refractory prostate cancer; *primaquine* and *pyrimethamine*, two anti-parasitic drugs; *trimethoprim*, an antibiotic; and *gliclazide*, an anti-diabetic drug (See Appendix A, titled ‘Repurposable Drugs for Treatment of Malignant Pleural Mesothelioma’). The drugs were selected based on whether they induced a differential expression (DE) in genes that showed a negative correlation with lung cancer associated DE, and affected genes of high DE in MPM tumors/cell lines (GSE51024 [33] and GSE2549 [34]), or underwent prior clinical testing in lung cancer. Lung cancers share common pathways with mesothelioma initiated on asbestos exposure. Therefore, drugs targeting lung cancers can potentially be used in MPM [35]. Table 3 shows pharmacokinetic details of the drugs as reported in Drug Bank [36].

Although in each case, there would be some genes that are differentially expressed in the same direction for both the drug and the disorder (for e.g., both the drug and the disease cause some genes to overexpress), the overall effect on the entire transcriptome has an anti-correlation. A correlation score is generated based on the strength of the overlap between the drug and the disease datasets. Statistical criteria such as correction for multiple hypothesis testing are applied and the correlated datasets are then ranked by statistical significance. A numerical score of 100 is assigned to the most significant result, and the scores of the other results are normalized with respect to this top-ranked result. We excluded drugs with unacceptable toxicity (e.g., minocycline) or unsuitable pharmacokinetics. The final list comprised 15 drugs, out of which 10 have already been tested against mesothelioma in clinical trials/animal models, and several of them were found to display clinical activity [37,38,39,40,41,42,43,44,45,46,47,48,49,50,51,52,53] (Table S2). Gemcitabine and pemetrexed are being used as first-line therapy for mesothelioma, in combination with cisplatin [45,53]. Ipilimumab has been identified to be a potential second-line or third-line therapy in combination with nivolumab [47]. Ixabepilone stabilizes cancer progression for up to 28 months [49]. Zoledronate, which showed modest activity in MPM, induced apoptosis and S-phase arrest in human mesothelioma cells and inhibited tumor growth in an orthotopic animal model [54,55]. Sirolimus/cisplatin increased cell death and decreased cell proliferation in MPM cell lines [56]. α-Tocopheryl succinate increased the survival of orthotopic animal models of malignant peritoneal mesothelioma [57]. Pre-clinical testing of vitamin E and its analogs are in progress [58,59].

Primaquine targets *KRT7*, a novel interactor of *KRT5*, whose high expression has been correlated with tumour aggressiveness and drug resistance in malignant mesothelioma [60,61,62]. Primaquine may be re-purposed for MPM treatment at least as an adjunctive drug with pemetrexed, the drug currently used for first-line therapy. Primaquine enhanced the sensitivity of the multi-drug resistant cell line KBV20C to cancer drugs [63]. Gliclazide is an anti-diabetic drug inhibiting *VEGFA* [64], a known interactor of *KDR*, and is significantly upregulated in MPM tumour (Log_2_FC = 1.83, *p*-value = 0.0018). Glicazide inhibits VEGF-mediated neovascularization [64]. High levels of VEGF have been correlated with both asbestos exposure in MPM and advanced cancer [65,66]. Glibenclamide, a drug with a similar mechanism of action as that of glicazide, increases caspase activity in MPM cell lines and primary cultures, leading to apoptosis mediated by *TRAIL* (TNF-related apoptosis inducing ligand) [67].

Eliminating those drugs which are being/have already been tested in mesothelioma with varying results, we arrived at a list of five potentially repurposable drugs in the descending order of negative correlation scores: pyrimethamine, cabazitaxel, primaquine, trimethoprim and gliclazide (Table 3). Cabazitaxel targets the MPM genes, *TUBB1* and *TUBA4A*, and was effective in treating non-small cell lung cancer (NSCLC) that was resistant to docetaxel, a drug that targets *TUBB1* along with other known interactors of MPM genes [37]. Pyrimethamine and trimethoprim target the MPM gene *TYMS* involved in folate metabolism, which was found to be differentially expressed in MPM tumors (GSE51024 [33]) (log_2_FC = 1.82, *p*-value = 4.10 × 10^−17^). MPM tumors have been shown to be responsive to anti-folates [68].

### 2.6. Analysis with Other High-Throughput Data

This section describes the overlap of the MPM interactome with various types of MPM-related biological evidence. 1690 (85.65%) proteins in the interactome were supported by genetic variant, transcriptomic, and proteomic evidence, and are listed in Data File S5. Table 4 shows 48 novel interactors that had three or more pieces of biological evidence.

**Figure 6:**
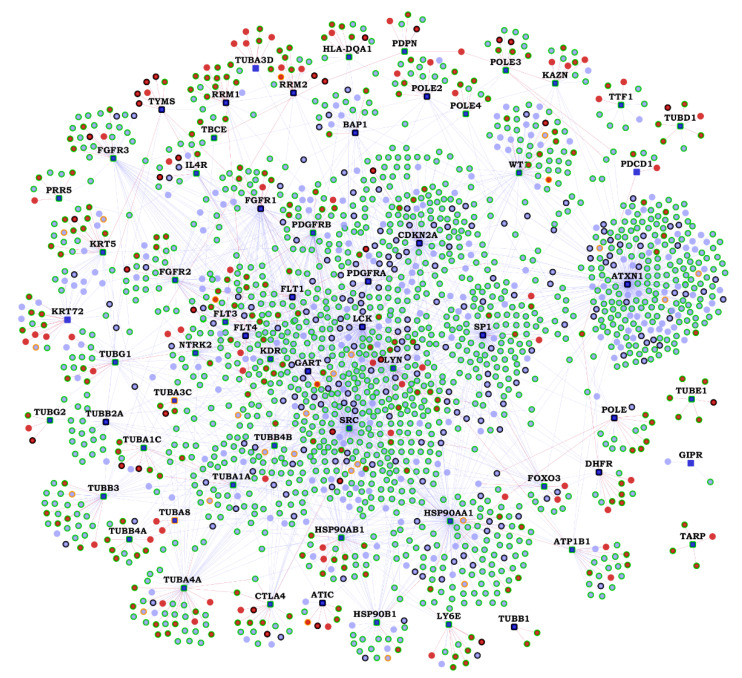
Genes with biological evidences in the malignant pleural mesothelioma (MPM) Protein-Protein Interactome: On the interactome network shown in Figure 1, various biological evidences of relation to malignant pleural mesothelioma (MPM) are shown as node border colors. Genes with variants associated with MPM have orange borders, genes with MPM/lung cancer/asbestos exposure-associated gene/protein expression changes have light green-colored borders and genes with black border have both genetic variants and gene/protein expression changes associated with them. The gene expression-associated features include differential expression in MPM tumors versus normal adjacent pleura, MPM tumors versus other thoracic tumors, differential gene methylation (affecting gene expression) in MPM tumors, gene expression correlated with unfavorable lung cancer prognosis, differential gene expression on exposure to asbestos or asbestos-like particles and high/medium expression in normal lungs. The protein expression-associated features include isolation as exosome-derived proteins from malignant mesothelioma cell lines and differential protein abundance levels in epithelioid and sarcomatoid types of malignant mesothelioma. The complete list of genes in the interactome and their corresponding evidence can be found in Data File S5.

We compiled the list of genes harboring MPM-associated genetic variants from Bueno et al. [5], and compared this list with all the genes in the MPM interactome (i.e., MPM-associated genes, their known and novel interactors) to identify overlaps. 275 genes in the MPM interactome harbored either germline mutations, or somatic single nucleotide variants (SNVs) or indels (insertions or deletions) (Figure 6, Table 4 and Data File S5) associated with MPM tumors. Of these 275 genes, 37 were novel interactors of MPM genes. *MGMT* carried germline mutations while the following carried somatic mutations: *ASTN2*, *BARX1*, *BRD2*, *CALML5*, *CAPRIN1*, *CLK1*, *CPS1*, *DPYD*, *EIF3H*, *EPB41L3*, *GMPS*, *GPR12*, *ITGAM*, *KIAA1524*, *KMT2D*, *KRT4*, *MGAT4A*, *NBR2*, *NDUFV2*, *NFIB*, *NFX1*, *NUDC*, *PLCL1*, *PRDM2*, *PRKAG1*, *PRMT1*, *PTPRT*, *PTRH2*, *RBBP6*, *SGK3*, *SLC20A1*, *SMCHD1*, *SPOCK1*, *TMPRSS15*, *TNC* and *XPO4*. Fourteen of these interact with MPM genes that also harbored a genetic variant (MPM genes are shown in bold): ***CDKN2A***-*NFX1*, ***FLT1***-*LATS2*, ***TUBA3C***-*XPO4*, ***PDGFRA***-*SPOCK1*, ***TYMS***-*SMCHD1*, ***TYMS***-*EPB41L3*, ***GART***-*TMPRSS15*, ***TYMS***-*NDUFV2*, ***TYMS***-*ITGAM*, ***RRM2***-*BARX1*, ***RRM2***-*MGAT4A* and ***ATIC***-*CPS1*, ***ATIC***-*KIAA1524* and ***POLE***-*NOS1*.

We collected the methylation profile of pleural mesothelioma [69], and found 8 novel interactors to be hypomethylated in pleural mesothelioma versus non-tumor pleural tissue, namely, *ACVR1B*, *IL6*, *MGMT*, *NRG1*, *OAT*, *PHLDA2*, *PLAUR* and *TNC* (Table S3). Some of them have little or no expression in lung tissue but are overexpressed in MPM. *PLAUR* is a prognostic biomarker of MPM [70]. Similarly, *FGFR1* and its novel interactor *NRG1* had elevated mRNA expression in H2722 mesothelioma cell lines and in MPM tissue, both contributing to increased cell growth under tumorigenic conditions [71,72]. *TNC*, involved in invasive growth, is a prognostic biomarker overexpressed in MPM, having low expression in normal lung tissues [73,74]. Thus, these novel interactors, which are not normally expressed in lung tissue, may be hypomethylated in MPM leading to their overexpression, contributing to MPM etiology.

Three hundred and ninety three (393) genes in the MPM interactome were also differentially expressed in mesothelioma tumors versus normal pleural tissue adjacent to tumor (GSE12345 [75]) (*p*-value of overlap = 9.525 × 10^−19^, odds ratio = 1.51). 52 out of the 314 novel interactors in the interactome were differentially expressed in this dataset (*p*-value = 0.046, odds ratio = 1.26). 938 genes, including 132 novel interactors, in the interactome were found to be differentially expressed in MPM tumors of epithelioid, biphasic and sarcomatoid types versus paired normal tissues (GSE51024 [33]) (*p*-value of overlap = 1.415 × 10^−18^, odds ratio = 1.24). Genes with fold-change >2 or <½ were considered as overexpressed and underexpressed, respectively, at a *p*-value < 0.05. Similarly, 744 genes in the MPM interactome were differentially expressed in MPM tumors versus other thoracic cancers such as thymoma and thyroid cancer (GSE42977 [76]) (*p*-value = 3.04 × 10^−41^, odds ratio = 1.53). 112 out of the 314 novel interactors in the interactome were differentially expressed in this dataset (*p*-value = 7.77 × 10^−6^, odds ratio = 1.45). This shows that the MPM interactome is enriched with genes whose expression helps in distinguishing MPM from other thoracic tumors and also with genes differentially expressed in mesothelioma tumors versus normal pleural tissue (Figure 6 and Data File S5). From RNA-seq data in GTEx, we found that 1311 genes, including 189 novel interactors, in the interactome have high/medium expression in normal lung tissue (median transcripts-per-million (TPM) > 9) (Figure 6 and Data File S5) [77].

A recent study had examined the gene expression profiles from the lungs of mice exposed to asbestos fibers (crocidolite and tremolite), an asbestiform fiber (erionite) and a mineral fiber (wollastonite) [78]. Crocidolite, tremolite and erionite are capable of inducing lung cancer and mesothelioma in humans and animal models [78]. On the other hand, wollastonite is a low pathogenicity fiber that shows no association with the incidence of lung cancer and mesothelioma in humans, or carcinogenesis in animal models [79]. The MPM interactome showed significant enrichment with all the 4 fibers (Figure 6 and Data File S5). The highest statistical significance was shown for the human orthologs of the mouse genes that were differentially expressed upon crocidolite exposure (199 genes, *p*-value = 1.16 × 10^−18^, odds ratio = 1.88). This was followed by tremolite (47 genes, *p*-value = 2.445 × 10^−5^, odds ratio = 1.87), wollastonite (16 genes, *p*-value = 0.0037, odds ratio = 2.09) and erionite (10 genes, *p*-value = 0.025, odds ratio = 2.01). Altogether, 245 genes in the interactome, including 29 novel interactors, have transcriptomic evidence with respect to exposure to asbestos or asbestos-like fibers. These novel interactors are: *ALB*, *B4GALT4*, *CAPN2*, *CDC40*, *DES*, *FMO1*, *FMR1*, *GML*, *GRIA1*, *HMG20B*, *HNRNPA1*, *ITSN2*, *LARP4*, *LPIN1*, *MGAT4A*, *NEK7*, *NFIB*, *NRG1*, *OCRL*, *PAX6*, *PDCD4*, *PITX3*, *PTRH2*, *REG3G*, *TAF1B*, *THOC1*, *TMED1*, *TNC* and *XPO4*.

From data in Pathology Atlas, we found that high expression of 73 genes, including that of 10 novel interactors, in the interactome has been positively correlated with unfavorable prognosis for lung cancer (*p*-value = 1.72 × 10^−9^, odds ratio = 2.05) [80]. These novel interactors are: *SPOCK1*, *SLC7A5*, *SCARB1*, *PLIN3*, *PLAUR*, *PIEZO1*, *KRT6A*, *GJB3*, *B3GNT3* and *ARL2BP*. We predicted *ARL2BP* to interact with *FLT1*, a VEGF receptor expressed in MPM cells. VEGF level in MPM patients is a biomarker for unfavorable prognosis, and lung cancer tumors expressing *FLT1* have been associated with poor prognosis [81,82].

Exosomes are extracellular vesicles secreted into the tumor microenvironment. They facilitate immunoregulation and metastasis by shuttling cellular cargo and directing intercellular communication. In a proteomic profiling study, 2176 proteins were identified in exosomes of at least one of the four human malignant mesothelioma cell lines (JO38, JU77, OLD1612 and LO68) [83]. 324 proteins in the MPM interactome appeared among these exosome-derived proteins (*p*-value = 8.86 × 10^−10^, odds ratio = 1.36), out of which 47 were novel interactors. Six hundred and thirty one (631) exosome-derived proteins were identified in all four malignant mesothelioma cell lines. Out of these, 127 occurred in the MPM interactome (*p*-value = 4.54 × 10^−12^, odds ratio = 1.84), out of which 15 were novel interactors (*PRKAG1*, *HNRNPA1*, *HNRNPH1*, *SORD*, *RNH1*, *RAN*, *PYGL*, *SLC7A5*, *RPS20*, *PARP4*, *YBX1*, *DCTN1*, *TUFM*, *EXOC4* and *GNPDA1*). In the following novel PPIs, both proteins involved in the interaction appeared among exosome-derived proteins (MPM gene in the interaction is shown in bold): ***TUBB3***-*SLC7A5*, ***HSP90AB1***-*PROS1*, ***HSP90AB1***-*GNPDA1*, ***TUBB4A***-*PLIN3*, ***LYN***-*ARFGEF1*, ***HSP90AA1***-*PHLDA2*, ***HSP90AA1***-*TCIRG1*, ***TUBG1***-*PHB*, ***GART***-*NMI*, ***SRC***-*CUL4B* and ***ATIC***-*CPS1*.

We computed the overlap of the interactome with 142 proteins that showed significant differences in abundance levels between epithelioid and sarcomatoid types of diffuse malignant mesothelioma [84]. In that study, a Fourier transform infrared (FTIR) imaging approach was employed to identify pathologic regions from diffuse malignant mesothelioma tissue samples [84]. These pathologic regions were then harvested using laser capture microdissection for proteomic analysis. 32 proteins in the interactome were more abundant in either epithelioid or sarcomatoid subtypes (*p*-value = 5.16 × 10^−5^, odds ratio = 2.06), including six novel interactors (*p*-value = 0.038, odds ratio = 2.43). The novel interactors *KRT78*, *NDUFV2*, *PRMT1*, *RAN* and *RNH1*—predicted to interact with the MPM genes *KRT72*, *TYMS*, *PDPN*, *POLE* and *RRM1*, respectively—had higher abundance in epithelioid samples, whereas *IGHA2*—predicted to interact with *HSP90AA1*—had higher abundance in sarcomatoid samples. The predicted interactions of these protein biomarkers with MPM-associated genes provide a mechanistic basis for experimental dissection of their ability to act as factors differentiating epithelioid tumors from sarcomatoid tumors (and vice versa).

## 3. Discussion

Currently, mesothelioma biologists only study a handful of genes, such as *BAP1*, *CDKN2A* and *NF2*. To shed light onto the other MPM-associated genes, whose functions remain poorly characterized, we assembled the ‘MPM interactome’ with ~2400 previously known PPIs and 364 computationally predicted PPIs (five of which have been validated in this work), which along with their biological annotations are being made available to researchers. We demonstrate the power of interactome-scale analyses to generate biologically insightful and clinically translatable results. The interactome has highly significant overlaps with MPM-associated genetic variants, genes differentially expressed or methylated in MPM or upon asbestos exposure, genes whose expression has been correlated with lung cancer prognosis, and with exosome-derived proteins in malignant mesothelioma cell lines. The interactome was enriched in cancer-related pathways. We extended the MPM interactome to include the drugs that target any of its proteins and analyzed it to identify a shortlist of 5 drugs that can potentially be repurposed for MPM—an example of a clinically translatable result.

We validated in vitro five novel PPIs in the interactome, namely, *BAP1*-*PARP3*, *ALB*-*KDR*, *ALB*-*PDGFRA*, *CUTA*-*HMGB1* and *CUTA*-*CLPS*. Literature evidence shows that these PPIs may be viable candidates for further experimentation in MPM cell lines or animal models. We hypothesize that the *BAP1*-*PARP3* interaction may enhance cancer growth in MPM. *BAP1* is a tumor suppressor protein playing a role in cell cycle progression, repair of DNA breaks, chromatin remodeling, and gene expression regulation; variants in *BAP1* have been implicated in hereditary and sporadic mesothelioma [85]. *PARP3* is involved in DNA repair, regulation of apoptosis, and maintenance of genomic stability and telomere integrity [86]. Interaction of *BAP1* with *BRCA1* has been shown to inhibit breast cancer growth [7]. In the absence of *BRCA1* activity or with a perturbation in its interaction with *BAP1*, cancerous growth is enhanced [87]. Loss of *BRCA1* protein expression has been noted in MPM [12]. In this scenario, it is possible that the novel interaction of *BAP1* with *PARP3* in cancerous cells may be promoting cancerous growth, possibly through regulation of DNA repair and apoptosis. *BAP1* and *PARP3* were found to be moderately overexpressed in sarcomatoid MPM tumors compared with normal pleural tissue (log_2_FC = 0.575, *p*-value = 0.028, and log_2_FC = 0.695, *p*-value = 0.0212, respectively) (GSE42977 [76]). Perturbation of the interaction of *BAP1* with *PARP3*, using *PARP3* inhibitors, may then suppress cancerous growth, at least in sarcomatoid MPM. Several studies and clinical trials [87], have shown that PARP inhibitors influence cancers in which mutations in *BRCA1* or *BRCA2* are observed, which led us to assume that the cancerous growth-inhibiting interaction of *BAP1* with *BRCA1* may already be perturbed in this case, and that PARP inhibitors may actually be blocking the novel interaction of *BAP1* with *PARP3* which enhances cancer growth. It has been pointed out that upon inhibiting PARP activity, cancerous cells that lack *BRCA1* or *BRCA2* activity may undergo cell cycle arrest and apoptosis, possibly due to an accumulation of chromatid aberrations and an inability to perform DNA repair in the absence of BRCA [7,87]. Thus, we suspect that the novel interaction of *BAP1* and *PARP3* may also be perturbed by PARP inhibitors, leading to inhibition of cancer growth.

Low levels of *ALB* have been correlated with poor prognosis in MPM patients [88]. The two MPM genes, *KDR* and *PDGFRA*, that *ALB* is predicted to interact with, are members of the PI3K/AKT pathway which has been shown to be aberrantly active in mesothelioma [89]. High expression of *CUTA* has been correlated with favorable prognosis in lung cancer (Pathology Atlas). It was found to be overexpressed in MPM tumors versus normal pleura (log_2_FC = 0.871, *p*-value = 0.0039) (GSE2549 [34]). *CLPS* inhibits metastasis of the melanoma cell line, B16F10, to lungs by blocking the signaling pathway involving β1 integrin, *FAK* and paxillin [90]. *CLPS* has a novel interaction with *NEDD9*, which has been shown to mediate β1 integrin signaling and promote metastasis of non-small lung cancer cells [91]. *CD26*, a cancer stem cell marker of malignant mesothelioma, has been shown to associate with the integrin α5β1 (or *ITGA5*, a novel interactor of the MPM gene, *FGFR2*) and promote cell migration and invasion in mesothelioma cells [91]. Another cancer stem cell marker of malignant mesothelioma, *CD9*, inhibits this metastatic effect mediated by *CD26*. Depletion of *CD26* and *CD9* was shown to respectively lead to decreased and increased expression of *NEDD9* and *FAK* in mesothelioma cells lines, hinting at the involvement of *NEDD9* in mesothelioma tumor invasiveness [91]. *NEDD9* has a known interaction with *LYN*, an MPM gene, shown to play a negative role in the regulation of integrin signaling in neutrophils [92]. *CUTA* has a novel interaction with *HMGB1*, which has been shown to activate the integrin αMβ2 (or *ITGAM*, a novel interactor of the MPM gene, *TYMS*) and the cell adhesion and migratory function of neutrophils mediated by αMβ2 [93]. *HMGB1* also has a novel interaction with the MPM gene, *FLT1*, shown to be involved in the migration of multiple myeloma cells by associating with β1 integrin, and mediating PKC activation [94].

A recent bioinformatics study identified the genes differentially expressed in epithelioid MPM tissues versus normal pleural tissues (GSE42977 [76]), and extracted the known PPIs interconnecting these genes from the STRING database [95]. They identified 10 hub genes from this network and shortlisted 31 drugs targeting the proteins in the network based on scores from the Drug-Gene Interaction Database (DGIdb). The DGIdb score takes into account the literature evidence for a particular drug-protein interaction, the number of proteins in the network that interact with the given drug, and the ratio of the average number of known protein interactors for all drugs compared to the number of known protein interactors for the given drug. *CDK1*, which is one of the hub genes identified in their study, is a known interactor of three MPM-associated genes, namely, *LYN*, *SP1* and *RRM2*, and we showed that it has association to MPM in three omics datasets: high expression correlated with unfavorable lung cancer prognosis, differential expression in MPM tumors versus adjacent pleural tissue, and isolation as an exosome-derived protein in malignant mesothelioma cell lines. Our work overall presents a more comprehensive study in terms of a larger number of MPM genes analyzed, which were compiled from multiple sources by IPA, and analysis of a larger number of MPM associated omics data sets, and presents transcriptomic-driven shortlisting of repurposable drugs for which additional evidence is presented from clinical trial data, literature, and differential expression of target genes in MPM datasets.

Our study provides an integrative and mechanistic framework for functional translation of mesothelioma-related multi-omics data. The novelty of our work stems from two key factors: (a) we present computationally predicted PPIs of high precision, which link MPM-related genes from disparate genetic-variant / transcriptomic/proteomic studies in hitherto unknown ways within the functional landscape of the interactome, and (b) the richly annotated MPM interactome is made available on a webserver to facilitate analysis by biologists and computational systems biologists. Our approach has some limitations. The drug-associated expression profiles analyzed in this study were induced in a diverse set of cell lines rather than in mesothelioma cell lines. The effect of the proposed drugs should be examined in MPM cell lines or animal models. We reported the overlap of mouse genes differentially expressed upon asbestos exposure [78] with corresponding human orthologs in the interactome. Mouse models have been routinely used to study pathologic changes associated with asbestos exposure, including gene expression, and these findings have been extrapolated to human diseases such as mesothelioma [96,97,98,99]. Nevertheless, our results should be interpreted with caution. It is not possible to draw direct transcriptomic/proteomic/phenotypic equivalences between mice and humans, unless these levels are comprehensively characterized in both the species, and a clear equivalence of factors defining a condition such as asbestos exposure is demonstrated in both the species [100]. Next, it is beyond the scope of our expertise to validate the large number of computationally predicted PPIs in a tissue or cell line of interest. However, we demonstrated the validity of computational predictions on a small number of PPIs on purified proteins with appropriate controls. The computational model has also been validated through additional experiments previously; some of the novel PPIs predicted previously by our method have translated into results of biomedical significance [17,18,19].

## 4. Methods

### 4.1. Data Collection

A search using the keyword “malignant pleural mesothelioma” on IPA (Ingenuity Pathway Analysis) retrieved genes causally related to the disease. IPA retrieves genes from the Ingenuity Knowledge Base which has ~5 million experimental findings expert-curated from biomedical literature or incorporated from other databases [29].

### 4.2. High-Precision Protein-Protein Interaction Prediction (HiPPIP) Model

PPIs were predicted by computing features of protein pairs, namely, cellular localization, molecular function and biological process membership, genomic location of the gene, gene expression from microarray experiments, protein domains and tissue membership of proteins, as described in Thahir et al. [101], and developing a random forest model to classify the pairwise features as interacting or non-interacting. A random forest with 30 trees was trained using the feature offering maximum information gain out of four random features to split each node; minimum number of samples in each leaf node was set to be 10. The random forest outputs a continuous valued score in the range of [0,1]. The threshold to assign a final label was varied over the range of the score for positive class (i.e., 0 to 1) to find the precision and recall combinations that are observed.

### 4.3. Evaluation of PPI Prediction Model

Evaluations on a held-out test data showed a precision of 97.5% and a recall of 5% at a threshold of 0.75 on the output score. Next, we created ranked lists for each of the hub genes (i.e., genes that had >50 known PPIs), where we considered all pairs that received a score >0.5 to be novel interactions. The predicted interactions of each of the hub genes are arranged in descending order of the prediction score, and precision versus recall is computed by varying the threshold of predicted score from 1 to 0. Next, by scanning these ranked lists from top to bottom, the number of true positives versus false positives was computed.

### 4.4. Novel PPIs in the MPM Interactome

Each MPM gene, say Z, is paired with each of the other human genes (G_1_, G_2_ … G_N_), and each pair is evaluated with the HiPPIP model. The predicted interactions of each of the MPM genes (namely, the pairs whose score is >0.5) were extracted. These PPIs, combined with the previously known PPIs of MPM genes collectively form the ‘MPM interactome’. Interactome figures were created using Cytoscape [102].

Note that 0.5 is the threshold chosen not because it is the midpoint between the two classes, but because the evaluations with hub proteins showed that the pairs that received a score >0.5 are highly confident to be interacting pairs. This was further validated through experiments for a few novel PPIs above this score.

### 4.5. Previously Known PPIs in the MPM Interactome

Previously known PPIs of the 62 MPM genes were collected from Human Protein Reference Database (HPRD) version 9 [22] and Biological General Repository for Interaction Datasets (BioGRID) version 4.3.194 [23]. The data behind our web-server will be updated once in a year with recent versions of BioGRID, and if novel PPIs are shown validated by such updates to known PPIs, the information will be posted on the web-server.

### 4.6. In Vitro Pull-Down Assays

An initial screening to find physical interactions was performed using an in vitro pull-down assay for some of the predicted novel PPIs. This technique utilizes a His/biotin tag-fused protein immobilized on an affinity column as the bait protein and a passing-through solution containing the ‘prey’ protein that binds to the ‘bait’ protein. The subsequent elution will pull down both the target (prey) and tagged-protein (bait) for further analysis by immunoblotting to confirm the predicted interactions. The pull-down assays were conducted using the Pull-Down PolyHis Protein:Protein Interaction Kit (Pierce^™^_,_ Rockford, IL, USA) according to the manufacturer’s instructions.

### 4.7. Protein Identification Methods

Peptide sequencing experiments were performed using an EASY-nLC 1000 coupled to a Q Exactive Orbitrap Mass Spectrometer (Thermo Scientific, San Jose, CA, USA) operating in positive ion mode. An EasySpray C18 column (2 µm particle size, 75 µm diameter by 15 cm length) was loaded with 500 ng of protein digest in 22 µL of solvent A (water, 0.1% formic acid) at a pressure of 800 bar. Separations were performed using a linear gradient ramping from 5% solvent B (75% acetonitrile, 25% water, 0.1% formic acid) to 30% solvent B over 120 min, flowing at 300 nL/min.

The mass spectrometer was operated in data-dependent acquisition mode. Precursor scans were acquired at 70,000 resolution over 300–1750 m/z mass range (3e6 AGC target, 20 ms maximum injection time). Tandem MS spectra were acquired using HCD of the top 10 most abundant precursor ions at 17,500 resolution (NCE 28, 1e5 AGC target, 60 ms maximum injection time, 2.0 *m/z* isolation window). Charge states 1, 6–8 and higher were excluded for fragmentation and dynamic exclusion was set to 20.0 s.

Mass spectra were searched for peptide identifications using Proteome Discoverer 2.1 (Thermo Scientific, Waltham, MA, USA) using the Sequest HT and MSAmanda algorithms, peptide spectral matches were validated using Percolator (target FDR 1%). Initial searches were performed against the complete UniProt database (downloaded 19 March 2018). Peptide matches were restricted to 10 ppm MS1 tolerance, 20 mmu MS2 tolerance, and 2 missed tryptic cleavages. Fixed modifications were limited to cysteine carbamidomethylation, and dynamic modifications were methionine oxidation and protein N-terminal acetylation. Peptide and protein grouping and results validation was performed using Scaffold 4.8.4 (Proteome Software, Portland, OR, USA) along with the X! Tandem algorithm against the previously described database. Proteins were filtered using a 99% FDR threshold.

### 4.8. Ingenuity Pathway Analysis

Pathway associations of genes in the MPM interactome were computed using Ingenuity Pathway Analysis (IPA). Statistical significance of the overlaps between genes in the MPM interactome and pathways in the Ingenuity Knowledge Base (IKB) was computed with Fisher’s exact test based on hypergeometric distribution. In this method, *p*-value is computed from the probability of k successes in n draws (without replacement) from a finite population of size N containing exactly k objects with an interesting feature, where N = total number of genes associated with pathways in IKB, K = number of genes associated with a particular pathway in IKB, n = number of genes in the MPM interactome and k = K ∩ n. This value was further adjusted for multiple hypothesis correction using the Benjamini-Hochberg procedure.

### 4.9. Analysis of Differential Gene Expression in Pleural Mesothelioma Tumors and Lungs of Asbestos-Exposed Mice Versus Normal Tissue in Lungs

The overlap of the MPM interactome with genes differentially expressed in pleural mesothelioma tumors compared with normal pleural tissue adjacent to mesothelioma was computed using the dataset GSE12345 [75]. Genes differentially expressed in the lungs of mice exposed to crocidolite and erionite fibers were obtained from the dataset GSE100900 [78]. Genes with fold change >2 or ½ were considered as significantly overexpressed and underexpressed respectively at *p*-value < 0.05.

### 4.10. Analysis of DNA Methylation in MPM Tumors

The dataset GSE16559 [69] was used to analyze the methylation profile of pleural mesotheliomas. In this study, genes found to be differentially methylated in mesothelioma were identified from a set of 773 cancer-related genes associated with 1413 autosomal CpG loci. Methylation values (M-values) were computed as M = log2 (β (1−β)) for both control (non-tumor pleural tissue) and test (pleural mesothelioma) cases, where β is the ratio of methylated probe intensity and overall intensity. Difference between M-values of test and control cases was then computed, and genes with M-value > 1 and M-value < 1 were considered to be hypermethylated and hypomethylated respectively at *p*-value < 0.05.

### 4.11. Correlating Expression of MPM Genes with Lung Cancer Prognosis

Data for correlation of gene expression and fraction of patient population surviving after treatment for lung cancer was taken from the Pathology Atlas [80]. Genes with log-rank *p*-value < 0.001 were considered to be prognostic. Unfavorable prognosis indicates positive correlation of high gene expression with reduced patient survival.

### 4.12. Identification of Repurposable Drugs in the MPM Drug-Protein Interactome

Negative correlation between lung cancer and drugs were studied using the BaseSpace correlation software, which uses a non-parametric rank-based approach to compute the extent of enrichment of a particular set of genes (or ‘bioset’) in another set of genes [31]. Readers may refer to Appendix A, titled ‘Repurposable Drugs for Treatment of Malignant Pleural Mesothelioma (MPM)’ for more details on the methodology used to identify repurposable drugs.

## 5. Conclusions

Biomedical discovery in the field of MPM research has to be accelerated to fuel clinically translatable results due to an urgent need to diagnose MPM preemptively, prevent its post-treatment recurrence, and curb its predicted increase in incidence in western and economically emerging nations [103]. In this study, we presented the MPM interactome as a valuable resource for mesothelioma biologists. We demonstrated its biological validity through comparison with MPM-related multi-omics data, which served to contextualize the novel PPIs within the mesothelioma landscape. Making novel MPM PPIs available freely on a webserver will catalyze investigations into these by cancer biologists and may lead to biologically or clinically translatable results. The MPM interactome with disease-associated proteins and their interacting partners will help biologists, bioinformaticians and clinicians to piece together an integrated view on how MPM-associated genes from various studies are functionally linked. Biological insights from this ‘systems-level’ view will help generate testable hypotheses and clinically translatable results. Future work will focus on expanding this interactome by including interactions from additional PPI repositories, other mesothelioma types and mesothelioma datasets.

## Figures and Tables

**Table 1:** Novel Interactors of each of the malignant pleural mesothelioma (MPM) Genes: Number of known (K) and computationally predicted novel (N) protein-protein interactions (PPIs) and lists the novel interactors. Bold genes in the 4th column are Novel Interactors that were experimentally validated in the current study.

Gene	K	N	Novel Interactors
*ATP1B1*	21	7	*HCRTR1, SERPINC1, TM4SF1, PRRX1, CD84, CREG1, THOC1*
*ATIC*	5	5	*MAP3K7, CPS1, KIAA1524, VWC2L, DES*
*ATXN1*	287	5	*CNOT6L, XPO7, C7, PITX3, RPL19*
*BAP1*	27	2	*PLN, **PARP3***
*CDKN2A*	168	5	*NFX1, DNAI1, GLIPR2, SIT1, CA9*
*CTLA4*	17	10	*PLCL1, DCTD, SKP1, GLP1R, AOX1, CD28, ATP5G3, CLK1, BCS1L, CDC26*
*DHFR*	10	7	*RHOQ, SCZD1, TOMM7, EXOC4, DTYMK, COPS8, CRHBP*
*FGFR1*	67	7	*ZFYVE1, NRG1, TPMT, OR51B4, SHB, PPP2CB, EIF4EBP1*
*FGFR2*	46	8	*PTPRE, OAT, PLXNA1, SEC23IP, MDM2, MGMT, PLSCR1, ELK4*
*FGFR3*	43	6	*GRK4, GMPS, STK32B, IDUA, IRF2BPL, ADD1*
*FLT1*	25	8	*MIPEP, RASSF9, HMGB1, FLT3, LATS2, ALOX5AP, ARL2BP, CDK8*
*FLT3*	17	8	*FMO1, SNRPA1, PNPLA3, NFIB, GPR12, SHC1, FLT1, CDK8*
*FLT4*	16	4	*NKX2-5, HNRNPH1, GRIA1, PNPLA8*
*FOXO3*	27	4	*GPR6, HDAC2, PRDM13, SIM1*
*GART*	4	5	*TIAM1, NMI, TMPRSS15, JUN, IFNAR1*
*GIPR*	2	0	*None*
*HLA-DQA1*	9	6	*HLA-DQA2, KLHDC3, TAL2, NXF1, BRD2, HLA-DPB1*
*HSP90AA1*	158	6	*IGHA2, MED28, PHLDA2, TCIRG1, IGHD, USP13*
*HSP90AB1*	59	10	*SLC25A27, PENK, ZFP36L2, MTX2, TPSAB1, PROS1, GPRC5B, CCR7, GNPDA1, CETN3*
*HSP90B1*	36	2	*MMP17, EPB41L4B*
*IL4R*	23	5	*RBBP6, NPIPB5, SLC20A1, ERN2, HDGFRP3*
*KAZN*	12	6	*KIF1B, NPPA, CELA2A, CELA2B, CTRC, FBLIM1*
*KDR*	60	8	*UTP3, SRP72, SHOX2, KIT, **ALB**, CACNA1S, CHIC2, GSTA2*
*KRT5*	25	10	*SORD, KRT6A, NADSYN1, SAP18, KRT7, TARBP2, KRT6B, KRT4, DCTN1, GPD1*
*KRT72*	19	8	*SP7, KRT78, KRT80, LARP4, MYL6B, KRT74, BCDIN3D, GRASP*
*LCK*	143	5	*NCDN, ZSCAN20, YBX1, CITED4, CAMK1D*
*LY6E*	6	8	*PIP, GLI4, HSF1, AKR1B1, EIF3H, JRK, GML, GPAA1*
*LYN*	125	12	*NEK7, SGK3, PDCD4, TRPA1, TERF1, PNMA2, IL7, CLCF1, AGXT, ARFGEF1, CRH, KLHL41*
*NTRK2*	34	3	*NXNL2, KCNS1, CDK20*
*PDCD1*	2	3	*COPS8, MCL1, OR6B3*
*PDGFRA*	64	4	*SPOCK1, RAPGEF1, **ALB**, CD244*
*PDGFRB*	76	8	*PLAUR, TUFM, CDX1, CHRM3, FAXDC2, ITK, CDK14, MITF*
*PDPN*	2	5	*PRDM2, PRMT1, ZBTB48, CELA2B, LHX1*
*POLE*	12	7	*SCARB1, RAN, VSIG4, ULK1, EIF2B1, MMP17, NOS1*
*POLE2*	19	6	*SAV1, PYGL, NID2, PARK7, DRD3, ATOH1*
*POLE3*	7	7	*TNC, TRIM32, EIF4G2, ASTN2, GSN, CST3, ALAD*
*POLE4*	7	4	*REG3G, SGOL1, EVA1A, B4GALT4*
*PRR5*	5	3	*WNT7B, TTC38, SCUBE1*
*RRM1*	10	12	*SLC22A18AS, SIRPA, SLC22A18, STIM1, SPINK1, ZFPM2, SH2D3A, PSMD13, RNH1, NUP98, CUZD1, RGS4*
*RRM2*	9	10	*TAF1B, ST3GAL3, NPBWR2, LPIN1, GCG, MGAT4A, BARX1, ASAP2, ITSN2, LAPTM4A*
*SP1*	146	5	*HNRNPA1, REG1A, RAPGEF3, GRIN1, ENDOU*
*SRC*	300	9	*ZNF687, ENPP7, FMR1, PI3, PTPRT, CUL4B, DPYD, BARD1, PLTP*
*TARP*	1	4	*TBX20, GGCT, IL6, CPVL*
*TBCE*	2	3	*SERTAD3, EIF2B2, PRDM2*
*TTF1*	6	3	*AMPH, DFNB31, QRFP*
*TUBA1A*	63	3	*TUBA1C, AMHR2, ACVR1B*
*TUBA1C*	63	8	*PRKAG1, SHMT2, AMHR2, SCAF11, ACVR1B, AQP5, KMT2D, TUBA1A*
*TUBA3C*	12	3	*XPO4, EIF3FP2, PARP4*
*TUBA3D*	1	6	*TUBA3E, WTH3DI, CCDC74B, FAM168B, LOC151121, IMP4*
*TUBA4A*	51	14	*WNT6, ETV6, ATP5G3, CAPN2, CXCR1, SLC11A1, CDK5R2, ALPP, IL1RL1, NUPR1, HPCA, SKP1, DPYSL2, STK16*
*TUBA8*	7	2	*POTEH, CCT8L2*
*TUBB1*	1	2	*C20orf85, SLMO2*
*TUBB2A*	27	0	*None*
*TUBB3*	34	6	*PRDM7, SLC7A5, PIEZO1, MVD, TRAPPC2L, TCF25*
*TUBB4A*	10	7	*UQCR11, APC2, ABCA7, PLIN3, KDM4B, SBNO2, HMG20B*
*TUBB4B*	19	4	*TSC1, NELFB, C9orf9, PTPRE*
*TUBD1*	1	6	*TMED1, PTRH2, TRPV1, GJB3, EPX, RFX5*
*TUBE1*	0	6	*DPAGT1, NUDC, RPS20, CDC40, GOPC, C6orf203*
*TUBG1*	28	6	*WNT3, PHB, RND2, CTRL, SGCA, RARA*
*TUBG2*	3	3	*NBR2, IKZF3, CLMP*
*TYMS*	3	9	*YES1, TAF3, ITGAM, NDUFV2, EPB41L3, SMCHD1, OCRL, THOC1, NAPG*
*WT1*	64	8	*FJX1, PEX3, CAPRIN1, PAX6, BST2, B3GNT3, CALML5, HIPK3*

**Table 2:** Pathways that are relevant to the pathophysiology and genetics of malignant pleural mesothelioma: Pathway analysis revealed that molecular mechanisms underlying various types of cancers, axonal guidance signaling, PI3/AKT signaling, VEGF signaling, natural killer cell signaling and inflammation signaling pathways may be pertinent to the development of MPM. A list of all the associated pathways is shown in Data File S3.

Pathway	*p*-Value	MPM Genes	Novel Interactors
Glucocorticoid Receptor Signaling	6.13 × 10^−56^	*KRT72*, *HSP90B1*, *FGFR3*, *HSP90AB1*, *FGFR1*, *KRT5*, *FOXO3*, *FGFR2*, *HSP90AA1*	*KRT74*, *HMGB1*, *PRKAG1*, *IL6*, *KRT6B*, *KRT78, KRT80*, *KRT7*, *KRT4*, *TAF3*, *NPPA*, *MAP3K7*, *KRT6A*
Molecular Mechanisms of Cancer	5.01 × 10^−53^	*CDKN2A*, *SRC*, *FGFR3*, *FGFR1*, *FGFR2*	*CDK14*, *CDK20*, *CDKN2B*, *PRKAG1*, *WNT7B*, *RND2*, *WNT6*, *CDK8*, *RHOQ*, *RAPGEF3*, *MAP3K7*, *WNT3*
NF-κB Signaling	1.26 × 10^−39^	*FGFR1*, *LCK*, *FLT1*, *KDR*, *PDGFRA*, *FGFR2*, *NTRK2*, *FGFR3*, *PDGFRB*, *FLT4*	*MAP3K7*
Small Cell Lung Cancer Signaling	2.00 × 10^−37^	*FGFR1*, *FGFR2*, *FGFR3*	*CDKN2B*
Axonal Guidance Signaling	2.51 × 10^−37^	*TUBB1*, *TUBA1A*, *TUBA4A*, *TUBA8*, *TUBB2A*, *NTRK2*, *FGFR3*, *FGFR1*, *TUBB3*, *TUBG1*, *TUBA1C*, *TUBB4B*, *FGFR2*, *TUBB4A*	*MYL6B*, *DPYSL2*, *PRKAG1*, *PLCL1*, *WNT7B*, *WNT6*, *PLXNA1*, *TUBA3E*, *WNT3*
PI3K/AKT Signaling	1.58 × 10^−36^	*HSP90B1*, *FOXO3*, *HSP90AA1*, *HSP90AB1*	*OCRL*, *PPP2CB*, *MCL1*, *EIF4EBP1*
VEGF Signaling	3.98 × 10^−36^	*FGFR1*, *FLT1*, *SRC*, *KDR*, *FOXO3*, *FGFR2*, *FGFR3*, *FLT4*	*EIF2B1*, *EIF2B2*
Role of Macrophages, Fibroblasts and Endothelial Cells in Rheumatoid Arthritis	6.31 × 10^−36^	*SRC*, *FGFR3*, *FGFR1*, *FGFR2*	*IL1RL1*, *IL6*, *PLCL1*, *WNT7B*, *IL7*, *WNT6*, *CALML5*, *MAP3K7*, *WNT3*, *APC2*
Natural Killer Cell Signaling	6.31 × 10^−32^	*FGFR1*, *LCK*, *FGFR2*, *FGFR3*	*OCRL*, *CD244*
Actin Cytoskeleton Signaling	1.58 × 10^−30^	*FGFR1*, *FGFR2*, *FGFR3*	*MYL6B*, *GSN*, *APC2*
eNOS Signaling	3.16 × 10^−30^	*FGFR1*, *FLT1*, *KDR*, *HSP90B1*, *FGFR2*, *HSP90AA1*, *FGFR3*, *FLT4*, *HSP90AB1*	*PRKAG1*, *CALML5*, *AQP5*, *CHRM3*
Neuroinflammation Signaling Pathway	3.98 × 10^−30^	*FGFR1*, *HLA-DQA1*, *FGFR2*, *FGFR3*	*HMGB1*, *HLA-DQB1*, *ACVR1B*, *IL6*, *GRIN1*, *GRIA1*
Gap Junction Signaling	1.00 × 10^−29^	*FGFR1*, *TUBB3*, *TUBG1*, *TUBB1*, *TUBA1C*, *TUBA1A*, *SRC*, *TUBB4B*, *TUBA4A*, *FGFR2*, *TUBA8*, *TUBB2A*, *FGFR3*, *SP1*, *TUBB4A*	*GJB3*, *PRKAG1*, *TUBA3E*, *PLCL1*, *GRIA1*
Integrin Signaling	1.58 × 10^−28^	*FGFR1*, *SRC*, *FGFR2*, *FGFR3*	*GSN*, *ITGAM*, *RHOQ*, *CAPN2*, *RND2*
IL-6 Signaling	1.58 × 10^−28^	*FGFR1*, *FGFR2*, *FGFR3*	*IL1RL1*, *MCL1*, *IL6*, *MAP3K7*

**Table 3:** Pharmacokinetic details of known mesothelioma drugs and the drugs that are presented as candidates for repurposing. Known mesothelioma drugs are shown in bold italics. Score corresponds to scaled correlation score with lung cancer expression studies from BaseSpace (NextBio) analysis.

Drug Name & Score	Original Therapeutic Purpose(s)	Delivery	Half-Life	Toxicity	Targets
***Pemetrexed*** negative 79	Chemotherapeutic drug for pleural mesothelioma and non-small cell lung cancer	Powder for solution; Intravenous	3.5 h	Data not available	*ATIC*, *DHFR*, *GART*, *TYMS*
***Mitomycin*** negative 64	Chemotherapeutic drug for breast, bladder, esophageal, stomach, pancreas, mesothelioma, lung and liver cancers	Injection, powder or lyophilized for solution; Intravenous	8–48 min	Nausea and vomiting	-
Cabazitaxel negative 79	Anti-neoplastic agent in hormone-refractory metastatic prostate cancer	Solution; Intravenous	Rapid initial-phase of 4 min, intermediate-phase of 2 h and prolonged terminal-phase of 95 h	Neutropenia, hypersensitivity reactions, gastrointestinal symptoms, renal failure	*TUBB1*, *TUBA4A*
Pyrimethamine negative 83	Anti-parasitic agent in toxoplasmosis and acute malaria	Tablet; Oral	4 days	Data not available	*DHFR*
Trimethoprim negative 63	Anti-bacterial agent/antibiotic in urinary tract, respiratory tract and middle-ear infections and traveler’s diarrhea	Tablet/solution; Oral	8 to 11 h	Oral toxicity in mice at LD50 = 4850 mg/kg	*DHFR*, *TYMS*
Primaquine negative 71	Anti-malarial agent	Tablet; Oral	3.7 to 7.4 h	Data not available	*KRT7*
Gliclazide negative 56	Anti-diabetic/hypoglycemic medication in type 2 diabetes mellitus	Tablet; Oral	10.4 h	Oral toxicity in mice at LD50 = 3000 mg/kg, accumulation in people with severe hepatic and/or renal dysfunction, side-effects of hypoglycemia including dizziness, lack of energy, drowsiness, headache and sweating	*VEGFA*

**Table 4:** Novel interactors in the malignant pleural mesothelioma (MPM) interactome with biological evidences related to MPM. The table shows the following data in columns labeled A to F. (A) 48 novel interactors of MPM associated genes that have been linked to four or more biological evidences related to MPM, namely, **B1**: high or medium gene expression in lungs, **B2**: differential gene expression in MPM tumor versus other thoracic tumors, **B3**: differential gene expression in MPM tumor versus normal adjacent pleural tissue, **B4**: differential gene expression in MPM tumors of epithelioid, biphasic and sarcomatoid types, **B5**: differential gene methylation in MPM, **B6:** gene expression correlated with unfavorable lung cancer prognosis, **B7**: differential gene expression on exposure to asbestos or asbestos-like particles, **C**: isolation as exosome-derived proteins from malignant mesothelioma cell lines, **D**: differential protein abundance levels in epithelioid and sarcomatoid types of malignant mesothelioma, and **E**: genetic variants in MPM. Last column, **F**, gives the total number of sources of evidences for each gene. The complete list of biological evidence for all the genes in the interactome can be found in Data File S5.

A	B							C	D	E	F
Novel Interactor	Differential Gene Expression							Exosome-Derived Proteins	Differential Protein Levels	Genetic Variants	Total
	B1	B2	B3	B4	B5	B6	B7				
*CAPRIN1*	✓	✓	✓	✓				✓		✓	6
*RAN*	✓	✓	✓	✓				✓	✓		6
*TNC*	✓	✓			✓		✓	✓		✓	6
*CUL4B*	✓	✓	✓	✓				✓			5
*GMPS*	✓	✓	✓	✓						✓	5
*IL6*	✓	✓		✓	✓			✓			5
*MGMT*	✓	✓	✓		✓					✓	5
*NFIB*	✓		✓	✓			✓			✓	5
*NUDC*	✓	✓		✓				✓		✓	5
*PLAUR*	✓	✓		✓	✓	✓					5
*PLIN3*	✓	✓		✓		✓		✓			5
*PLXNA1*	✓	✓	✓	✓				✓			5
*PRMT1*	✓		✓					✓	✓	✓	5
*RNH1*	✓	✓		✓				✓	✓		5
*SCARB1*	✓	✓		✓		✓		✓			5
*SLC7A5*	✓		✓	✓		✓		✓			5
*SMCHD1*	✓		✓	✓				✓		✓	5
*ASAP2*	✓	✓		✓				✓			4
*B4GALT4*	✓	✓		✓			✓				4
*CAPN2*	✓	✓		✓			✓				4
*CDC40*	✓		✓	✓			✓				4
*DTYMK*	✓	✓	✓	✓							4
*EIF3H*	✓			✓				✓		✓	4
*EPB41L3*	✓	✓		✓						✓	4
*EXOC4*	✓	✓		✓				✓			4
*GNPDA1*	✓	✓		✓				✓			4
*HNRNPA1*	✓			✓			✓	✓			4
*HNRNPH1*	✓		✓	✓				✓			4
*LARP4*	✓		✓	✓			✓				4
*MGAT4A*	✓	✓					✓			✓	4
*MITF*	✓	✓	✓	✓							4
*NDUFV2*	✓	✓							✓	✓	4
*OAT*	✓	✓			✓			✓			4
*PHB*	✓	✓	✓					✓			4
*PHLDA2*	✓	✓			✓			✓			4
*PLCL1*		✓	✓	✓						✓	4
*PRKAG1*	✓	✓						✓		✓	4
*PROS1*	✓	✓		✓				✓			4
*PTRH2*		✓		✓			✓			✓	4
*PYGL*	✓	✓		✓				✓			4
*RBBP6*	✓		✓	✓						✓	4
*SEC23IP*	✓	✓	✓	✓							4
*SGK3*		✓	✓	✓						✓	4
*SHMT2*	✓	✓	✓	✓							4
*SLC20A1*	✓	✓		✓						✓	4
*TCIRG1*	✓	✓		✓				✓			4
*XPO4*				✓			✓	✓		✓	4
*YBX1*	✓	✓	✓					✓			4

## Data Availability

On journal website and at http://severus.dbmi.pitt.edu/wiki-MPM.

## Supplementary Materials

The following are available online at https://www.mdpi.com/article/10.3390/cancers13071660/s1: Table S1: Identification of protein interactors using liquid chromatography–mass spectrometry (LC-MS), Table S2: Overlaps between drugs tested in NSCLC and drugs occurring in the MPM drug-protein interactome, that were negatively correlated with lung cancer expression studies, Table S3: Some novel interactors which are hypomethylated and their MPM genes, Data File S1: List of MPM genes and their corresponding biological evidences extracted from IPA suite, Data File S2: List of genes from the MPM interactome with their labels (MPM genes, known interactors and novel interactors), Data File S3: List of all the pathways associated with at least one of the MPM genes, Data File S4: List of all the drugs that target any of the genes from the MPM interactome, and Data File S5: Master table of all biological evidences (genetic variant, transcriptomic and proteomic evidence) for each of the MPM interactome genes discussed in the paper.

## Appendix A. Repurposable Drugs for Treatment of Malignant Pleural Mesothelioma (MPM)

We present here five drugs (*cabazitaxel*, *pyrimethamine*, *trimethoprim*, *primaquine* and *glicazide*) that could potentially be repurposed for the treatment of malignant pleural mesothelioma (MPM). These drugs were shortlisted through three types of analysis: (A) considering those that were already tested in non-small cell lung cancer (NSCLC), (B) gene expression analysis of drugs that target MPM genes or novel interactors in the MPM interactome, or (C) gene expression analysis of drugs that target known interactors in the malignant pleural mesothelioma (MPM) interactome. Drugs were selected based on whether they were already tested against lung cancer in clinical trials and/or showed overall negative correlation with lung cancer expression studies, because both mesothelioma and lung cancers have been shown to share common pathways that are initiated on exposure to asbestos fibres in mesothelial cells and lung epithelial cells respectively [35]. Another criterion used was whether the genes targeted by the drugs showed high differential expression in MPM tumours/cell lines. The details of these methods and observations are presented below.

### Appendix A.1. Repurposable Drugs Already Tested in Non-Small Cell Lung Cancer

Nine overlapping drugs were found between drugs tested in NSCLC and drugs occurring in the MPM drug-protein interactome, that were negatively correlated with lung cancer expression studies, namely, cabazitaxel, dasatinib, docetaxel, gemcitabine, ipilimumab, ixabepilone, minocycline, pazopanib and pemetrexed. Minocycline was eliminated due to its toxicity. All of the remaining eight drugs were found to be effective in treatment of NSCLC (Table S2). Out of these eight drugs, cabazitaxel was the only drug that was not tested for treatment of mesothelioma. The fact that the other seven drugs were already tested against mesothelioma in clinical trials demonstrates the validity of our approach. It was interesting to note that drugs that targeted known interactors in addition to some MPM genes were found to have either no effect or limited clinical activity in mesothelioma, for e.g., dasatinib, docetaxel and pazopanib. On the other hand, drugs that targeted only MPM genes were found to be effective in treatment of mesothelioma or were capable of preventing disease progression, for e.g., gemcitabine, ipilimumab, ixabepilone and pemetrexed. This raises the suspicion that drugs that do not act on “off-target” genes (known interactors, in this case) may be more effective. In this respect, cabazitaxel, which targets the MPM genes *TUBB1* and *TUBA4A*, may be a suitable candidate for mesothelioma. Cabazitaxel was found to be effective in treatment of NSCLC resistant to docetaxel, a drug that targets *TUBB1* and other known interactors [37].

### Appendix A.2. Repurposable Drugs Targeting MPM Genes and Novel Interactors

The MPM genes that were most differentially expressed with high significance in MPM tumors (GSE51024 [33]) were *TYMS* (log_2_FC = 1.82, *p*-value = 4.10 × 10^−17^) and *DHFR* (log_2_FC = 0.89, *p*-value = 1.20 × 10^−14^), and the drugs that target these genes (also having negative correlation with lung cancer expression) were pyrimethamine and trimethoprim. The first line drug currently used to treat mesothelioma is premetrexed, which targets proteins in the folate metabolic pathway, namely, *DHFR*, *TYMS* and *GART* [104]. Since MPM tumors have been shown to be responsive to anti-folates [68], both pyrimethamine (which targets only *DHFR*) and trimethoprim (which targets both *DHFR* and *TYMS*), seem to be interesting candidates. Pyrimethamine, an anti-parasitic drug commonly used to treat toxoplasmosis and cystoisosporiasis, has shown anti-tumor activity in metastatic melanoma cells and in murine models of breast cancer [105,106]. Trimethoprim, an anti-bacterial drug commonly used in the treatment of urinary bladder and respiratory tract infections, is also used to treat bacterial infections in cancer patients [107,108].

Keratin proteins form important components of the cell cytoskeleton, called intermediate filaments, in epithelial cells, and are commonly used as diagnostic markers in cancer [60]. In addition to their role as cancer markers, their involvement in cellular functions such as cell motility, proliferation, cell polarity, protein synthesis, membrane trafficking and most importantly, tumour invasion and metastasis make them attractive as candidates for drug development [60]. *KRT7* is a keratin primarily expressed in mesothelial cells, apart from cells lining ducts and the intestine [60]. In a patient with malignant mesothelioma of the epithelioid type (which spreads to mediastinum and breast), *KRT7* was found to be significantly overexpressed when she developed resistance to pemetrexed/carboplatin, provided as a second line therapy [61]. The cancer cells showed a drastic increase in their immunoreactivity to CK7, the protein encoded by *KRT7* [61]. At the last stage of cancer progression (which was followed by her death), the patient showed dyspnoea (difficulty in breathing), increased tumour volume and pleural fluid [61]. In another case, *KRT7* was found be significantly overexpressed in an aggressive state of MPM, prior to treatment [61]. Two-thirds of malignant mesothelioma cases have been reported to be K7^+^/K20^−^ (positive for expression of *KRT7* and negative for expression of *KRT20*) [60]. Expression of *KRT7* in three histological types of mesothelioma, namely, epithelioid, sarcomatid and biphasic, has been used to distinguish them from synovial sarcoma that metastasizes to the lungs and pleura [62]. *KRT7* has been identified as marker of circulating tumour cells in lung cancer [109]. *KRT7* was also found to be significantly upregulated in MPM tumours (log_2_FC = 3.80, *p*-value = 0.0002), and in cell line models of MPM (log_2_FC = 2.266, *p*-value = 0.029) (GSE2549 [34]). Positive expression of *KRT7* was noted in various types of non-small cell lung cancers, including large cell neuroendocrine carcinoma and lung adenocarcinoma [110,111]. In the MPM interactome, *KRT7* was predicted to interact with *KRT5*, an MPM gene that serves as a marker for malignant mesothelioma, along with vimentin, and is specifically used to distinguish pleural mesothelioma of the epithelioid type from pulmonary adenocarcinoma and non-pulmonary adenocarcinoma metastasizing to pleura [60,112]. *KRT7* is a target of primaquine, an-antimalarial agent known to destroy the malarial parasites, *Plasmodium vivax* and *Plasmodium ovale*, inside the liver [113,114]. The exact mechanism of action has not been elucidated for this drug. However, in independent studies, primaquine has been shown to bind to keratin in a concentration-dependent manner, and also mediate strong membrane perturbations in cell membrane models [113,115]. Since high expression of *KRT7* has been shown to be related to tumour aggressiveness and drug resistance in malignant mesothelioma, and its high expression was also noted in MPM tumours and cell lines, primaquine may be re-purposed for treatment of MPM at least as an adjunctive drug with pemetrexed, the drug currently used for first line therapy. It is interesting to note that primaquine enhanced the sensitivity of KBV20C cells to cancer drugs, namely, vinblastine, vinorelbine, paclitaxel, docetaxel, vincristine and halaven [63]. KVB20C is a multi-drug resistant cell line derived from oral squamous carcinoma. Primaquine compounds (substituted quinolines) have also been shown to exert anti-tumor activity in breast cancer cells [116].

### Appendix A.3. Repurposable Drugs Targeting Known Interactors

Out of the four drugs targeting known interactors in the MPM interactome and showing negative correlation with lung cancer associated gene expression, three drugs were already known to exhibit anti-tumour activity in pre-clinical models of mesothelioma, namely, zoledronate, sirolimus and the vitamin E analog, alpha-tocopheryl succinate, which shows the validity of our approach. Zoledronate, which showed modest activity in MPM, induced apoptosis and S-phase arrest in human mesothelioma cells and inhibited tumor growth in the pleural cavity of an orthotopic animal model [54,55]. Sirolimus/cisplatin increased cell death and decreased cell proliferation in cell lines of MPM [56]. Alpha-tocopheryl succinate increased survival of orthotopic animal models of malignant peritoneal mesothelioma [57]. Zoledronate has demonstrated modest clinical activity in patients with advanced MPM [54]. Sirolimus has not been tested against MPM in clinical trials, but everolimus, a drug derived from sirolimus sharing similar properties with it, has shown only limited clinical activity in MPM, and further testing as a single-agent was not advised based on the results from this study [117]. Both vitamin E and its analog, alpha-tocopheryl succinate have not been tested against MPM in clinical trials. However, testing of vitamin E and its analogs are being carried out in various pre-clinical settings [58,59]. Hence, it was the drug gliclazide that emerged as a potentially repurposable drug, untested against MPM.

Gliclazide, an anti-diabetic drug, inhibits *VEGFA*, which has been shown to be significantly upregulated (Log_2_FC = 1.83, *p*-value = 0.0018) in MPM tumour (GSE2549 [34]). This drug inhibits VEGF expression induced by advanced glycation end products in bovine reticular endothelial cells, and VEGF expression induced by ischemia in retinal tissue of mice [64,118]. In the latter case, glicazide also inhibits neovascularization, a process known to be mediated by VEGF. VEGF has been identified as a prognostic marker for MPM. High levels of VEGF have been correlated with both asbestos exposure in MPM, and an advanced stage of the disease [65,66]. It is interesting to note that glibenclamide, a drug whose mechanism of action is similar to that of glicazide, has been shown to increase caspase activity in MPM cell lines and primary cultures, leading to apoptosis mediated by TNF-related apoptosis inducing ligand (*TRAIL*) [67]. Hence, glicazide may be repurposed to inhibit neovascularization and perhaps enhance apoptosis in MPM.
