# Supplementary material for: Malignant Pleural Mesothelioma Interactome with 364 Novel Protein-Protein Interactions
Source: Cancers (Basel). 2021 Apr 1;13(7):1660. doi: 10.3390/cancers13071660 (PMC8037232; doi:10.3390/cancers13071660)
Supplement: Supplementary file 1 [file cancers-13-01660-s001.zip › Supplementary Tables.docx]

**SUPPLEMENTARY TABLES**

**Table S1.** Identification of protein interactors using liquid chromatography–mass spectrometry (LC-MS)

| **Tested individual protein/protein pairs** | **Uniprot IDs protein(s)** | **Description of identified protein(s)** | **# Peptides** | **# AAs** | **MW**  **[kDa]** | **Peptide sequences detected** |
| --- | --- | --- | --- | --- | --- | --- |
| PLN | P26678 | Cardiac phospholamban OS=Homo sapiens GN=PLN PE=1 SV=1 - [PPLA_HUMAN] | 2 | 52 | 6.1 | 1. VQYLTRSA 2. RASTIEM PQQAR |
| BAP1+PLN  (Negative control) | Q92560 | Ubiquitin carboxyl-terminal hydrolase BAP1 OS=Homo sapiens GN=BAP1 PE=1 SV=2 - [BAP1_HUMAN] | 2 | 729 | 80.3 | 1.LPAFLDNHNYA  2.QGVSIG |
| BAP1+PARP3 | Q92560 | Ubiquitin carboxyl-terminal hydrolase BAP1 OS=Homo sapiens GN=BAP1 PE=1 SV=2 - [BAP1_HUMAN] | 2 | 729 | 80.3 | 1.LPAFLDNHNYA  2.QGVSIG |
|  | Q9Y6F1 | Poly [ADP-ribose] polymerase 3 OS=Homo sapiens GN=PARP3 PE=1 SV=3 - [PARP3_HUMAN] | 5 | 533 | 60 | 1.FYIIQLLQDSNR  2.DHFVSHPGKYTLI  3.NTMALMDLDVK  4.DMLLVLADIE  5.LAQALQAVSE QEK |
| CUTA | O60888 | Protein CutA OS=Homo sapiens GN=CUTA PE=1 SV=2 - [CUTA_HUMAN] | 3 | 179 | 19.1 | 1.LLLLPR  2.LAACVNLIPQITSIYEWK  3.IEEDSEVLMMIKTQSSLVP ALTDFV |
| CLPS+CUTA | O60888 | Protein CutA OS=Homo sapiens GN=CUTA PE=1 SV=2 - [CUTA_HUMAN] | 3 | 179 | 19.1 | 1.SRLLLLPRVLLTMASGSPPT QPSPASDSGSGYVPGSVSAA 2.LAACVNLIPQITSIYEWK  3.KIEEDSEVLMMIKTQSSLVP ALTDFVRSVHPYEVAEVIAL |
|  | P04118 | Colipase OS=Homo sapiens OX=9606 GN=CLPS PE=1 SV=2 | 3 | 112 | 11.9 | 1.GIIINLENGELCMNSAQCKS NCCQHSSALGLAR 2.GLTCEGDK TIVGSITNTN  3.FGICHDAGR |

**Table S2.** Overlaps between drugs tested in NSCLC and drugs occurring in the malignant pleural mesothelioma (MPM) drug-protein interactome, that were negatively correlated with lung cancer expression studies

| **Drug** | **Treatment outcome in NSCLC** | **Treatment outcome in mesothelioma** | **Targeted MPM genes** | **Targeted known interactors** |
| --- | --- | --- | --- | --- |
| Cabazitaxel | Effective in docetaxel-resistant NSCLC [1] | NOT TESTED | TUBB1, TUBA4A | - |
| Dasatinib | Modest activity, toxic effect: pleural effusions [2] | No effect, pulmonary toxicity [3] | LCK, PDGFRB | ABL1, FYN, YES1, KIT, STAT5B |
| Docetaxel | Effective with activity equivalent to standard therapy [4] | No effect as single agent, hemorrhage, gastrointestinal and pulmonary toxicity; effective when combined with gemcitabine [5-7] | TUBB1 | MAP2, MAPT, NR1I2 |
| Gemcitabine | First-line therapy in combination with cisplatin [8] | First-line therapy in combination with cisplatin [9] | RRM1, TYMS | - |
| Ipilimumab | Not effective as single agent, effective when combined with nivolumab [10] | Potential second- or third-line therapy in combination with nivolumab [11] | CTLA4 | - |
| Ixabepilone | Effective when combined with carboplatin [12] | Stable disease upto 28 months [13] | TUBB3 | - |
| Pazopanib | Effective, toxic effects: hypertension, diarrhea, and fatigue [14] | Limited effect, toxic effects: hypertension, proteinuria, liver enzyme elevations, myelosuppression, and fatigue [15] | FGFR3, FLT1, FLT4, KDR, PDGFRA, PDGFRB | FGF1, ITK, KIT |
| Pemetrexed | First-line therapy in combination with cisplatin [16] | First-line therapy in combination with cisplatin [17] | ATIC, DHFR, GART, TYMS | - |

**Table S3.** Some novel interactors which are hypomethylated and their malignant pleural mesothelioma (MPM) genes: CgTarget_id refers to accession key of CpG locus searchable on UCSC Browser, CpG_Coordinate is the position of the locus on the chromosome in base pairs and Dist_to_TSS is the distance of the CpG locus to the transcription start site of the gene. FGFR1-NRG1, PDGFRB-PLAUR and POLE3-TNC are novel interactions in the MPM interactome. Novel genes such as NRG1, PLAUR and TNC are hypomethylated, leading to their overexpression in MPM.

| **Node** | **Gene** | **CgTargetid** | **Chr** | **CpGCoordinate** | **Distance to TSS** | **Difference between M-values** | **P-value** |
| --- | --- | --- | --- | --- | --- | --- | --- |
| MPM gene | FGFR1 | cg05405947 | 8 | 38444976 | 317 | -1.310771249 | 2.35E-10 |
|  |  | cg20658205 | 8 | 38445497 | -204 | -1.250615821 | 5.81E-08 |
| Novel Interactor | NRG1 | cg25833018 | 8 | 32525369 | 74 | -1.136538266 | 1.44E-05 |
|  |  | cg12863621 | 8 | 32524737 | -558 | -1.129718887 | 1.61E-03 |
| MPM gene | PDGFRB | cg21817429 | 5 | 149515420 | 195 | -1.763499341 | 9.80E-12 |
|  |  | cg12727795 | 5 | 149515888 | -273 | -1.401530764 | 1.01E-12 |
| Novel Interactor | PLAUR | cg08793657 | 19 | 48866219 | 123 | -1.428731107 | 9.75E-10 |
|  |  | cg04828696 | 19 | 48866424 | -82 | -1.058287922 | 7.00E-03 |
| MPM gene | POLE3 | Not found in this dataset | | | | | |
| Novel Interactor | TNC | cg22480835 | 9 | 116920458 | -198 | -1.618947626 | 4.21E-09 |

**Supplementary References**

1. Kotsakis, A.; Matikas, A.; Koinis, F.; Kentepozidis, N.; Varthalitis, I.; Karavassilis, V.; Samantas, E.; Katsaounis, P.; Dermitzaki, E.; Hatzidaki, D. A multicentre phase II trial of cabazitaxel in patients with advanced non-small-cell lung cancer progressing after docetaxel-based chemotherapy. *British journal of cancer* **2016**, *115*, 784.

2. Johnson, F.M.; Bekele, B.N.; Feng, L.; Wistuba, I.; Tang, X.M.; Tran, H.T.; Erasmus, J.J.; Hwang, L.-L.; Takebe, N.; Blumenschein, G.R. Phase II study of dasatinib in patients with advanced non–small-cell lung cancer. *Journal of clinical oncology* **2010**, *28*, 4609-4615.

3. Tsao, A.S.; Lin, H.; Carter, B.W.; Lee, J.J.; Rice, D.; Vaporcyan, A.; Swisher, S.; Mehran, R.; Heymach, J.; Nilsson, M. Biomarker-Integrated Neoadjuvant Dasatinib Trial in Resectable Malignant Pleural Mesothelioma. *Journal of Thoracic Oncology* **2017**.

4. M Comer, A.; L Goa, K. *Docetaxel. A review of its use in non-small cell lung cancer*; 2000; Vol. 17, pp. 53-80.

5. Belani, C.P.; Adak, S.; Aisner, S.; Stella, P.J.; Levitan, N.; Johnson, D.H. Docetaxel for malignant mesothelioma: phase II study of the Eastern Cooperative Oncology Group. *Clinical lung cancer* **2004**, *6*, 43-47.

6. Ralli, M.; Tourkantonis, I.; Makrilia, N.; Gkini, E.; Kotteas, E.; Gkiozos, I.; Katirtzoglou, N.; Syrigos, K. Docetaxel plus gemcitabine as first-line treatment in malignant pleural mesothelioma: a single institution phase II study. *Anticancer research* **2009**, *29*, 3441-3444.

7. Tourkantonis, I.; Makrilia, N.; Ralli, M.; Alamara, C.; Nikolaidis, I.; Tsimpoukis, S.; Charpidou, A.; Kotanidou, A.; Syrigos, K. Phase II study of gemcitabine plus docetaxel as second-line treatment in malignant pleural mesothelioma: a single institution study. *American journal of clinical oncology* **2011**, *34*, 38-42.

8. Manegold, C. Gemcitabine (Gemzar®) in non-small cell lung cancer. *Expert review of anticancer therapy* **2004**, *4*, 345-360.

9. Kindler, H.L.; van Meerbeeck, J.P. The role of gemcitabine in the treatment of malignant mesothelioma. In Proceedings of Seminars in oncology; pp. 70-76.

10. Malhotra, J.; Jabbour, S.K.; Aisner, J. Current state of immunotherapy for non-small cell lung cancer. *Translational lung cancer research* **2017**, *6*, 196.

11. Scherpereel, A.; Mazieres, J.; Greillier, L.; Dô, P.; Bylicki, O.; Monnet, I.; Corre, R.; Audigier-Valette, C.; Locatelli-Sanchez, M.; Molinier, O. Second-or third-line nivolumab (Nivo) versus nivo plus ipilimumab (Ipi) in malignant pleural mesothelioma (MPM) patients: Results of the IFCT-1501 MAPS2 randomized phase II trial. American Society of Clinical Oncology: 2017.

12. Spigel, D.R.; Greco, F.A.; Waterhouse, D.M.; Shipley, D.L.; Zubkus, J.D.; Bury, M.J.; Webb, C.D.; Hart, L.L.; Gian, V.G.; Infante, J.R. Phase II trial of ixabepilone and carboplatin with or without bevacizumab in patients with previously untreated advanced non-small-cell lung cancer. *Lung Cancer* **2012**, *78*, 70-75.

13. Puhalla, S.; Brufsky, A. Ixabepilone: a new chemotherapeutic option for refractory metastatic breast cancer. *Biologics: targets & therapy* **2008**, *2*, 505.

14. Altorki, N.; Lane, M.E.; Bauer, T.; Lee, P.C.; Guarino, M.J.; Pass, H.; Felip, E.; Peylan-Ramu, N.; Gurpide, A.; Grannis, F.W. Phase II proof-of-concept study of pazopanib monotherapy in treatment-naive patients with stage I/II resectable non–small-cell lung cancer. *Journal of Clinical Oncology* **2010**, *28*, 3131-3137.

15. Hiddinga, B.I.; Rolfo, C.; van Meerbeeck, J.P. Mesothelioma treatment: Are we on target? A review. *Journal of advanced research* **2015**, *6*, 319-330.

16. Scagliotti, G.V.; Parikh, P.; Von Pawel, J.; Biesma, B.; Vansteenkiste, J.; Manegold, C.; Serwatowski, P.; Gatzemeier, U.; Digumarti, R.; Zukin, M. Phase III study comparing cisplatin plus gemcitabine with cisplatin plus pemetrexed in chemotherapy-naive patients with advanced-stage non–small-cell lung cancer. *Journal of clinical oncology* **2008**, *26*, 3543-3551.

17. Vogelzang, N.J.; Rusthoven, J.J.; Symanowski, J.; Denham, C.; Kaukel, E.; Ruffie, P.; Gatzemeier, U.; Boyer, M.; Emri, S.; Manegold, C. Phase III study of pemetrexed in combination with cisplatin versus cisplatin alone in patients with malignant pleural mesothelioma. *Journal of clinical oncology* **2003**, *21*, 2636-2644.
